# The effects of exercise and passive heating on the sweat glands ion reabsorption rates

**DOI:** 10.14814/phy2.13619

**Published:** 2018-02-27

**Authors:** Nicola Gerrett, Tatsuro Amano, Yoshimitsu Inoue, George Havenith, Narihiko Kondo

**Affiliations:** ^1^ Laboratory for Applied Human Physiology Graduate School of Human Development and Environment Kobe University Kobe Japan; ^2^ Laboratory for Exercise and Environmental Physiology Faculty of Education Niigata University Niigata Japan; ^3^ Laboratory for Human Performance Research Osaka International University Osaka Japan; ^4^ Environmental Ergonomics Research Centre Loughborough Design School Loughborough University Loughborough United Kingdom

**Keywords:** Endogenous vs. exogenous, regional differences, sweat ion regulation, sweating

## Abstract

The sweat glands maximum ion reabsorption rates were investigated (*n* = 12, 21.7 ± 3.0 years, 59.4 ± 9.8 kg, 166.9 ± 10.4 cm and 47.1 ± 7.5 mL/kg/min) during two separate endogenous protocols; cycling at 30% (LEX) and 60% *V*O_2max_ (MEX) and one exogenous trial; passive heating (PH) (43°C water lower leg immersion) in 27°C, 50%RH. Oesophageal temperature (*T*_es_), skin temperature (*T*_sk_), and forearm, chest and lower back sweat rate (SR) and galvanic skin conductance (GSC) were measured. Salivary aldosterone was measured pre‐and postheating (*n* = 3). Using the ∆SR threshold for an increasing ∆GSC to identify maximum sweat ion reabsorption rate revealed higher reabsorption rates during MEX compared to PH (mean of all regions: 0.63 ± 0.28 vs. 0.44 ± 0.3 mg/cm^2^/min, *P* < 0.05). It was not possible to identify the ion reabsorption rate during LEX for some participants. *T*_es_ and mean *T*_sk_ were different between conditions but mean body temperature (*T*_b_) and local *T*_sk_ (forearm, chest and back) were similar (*P* > 0.05). Aldosterone increased more during MEX (72.8 ± 36.6 pg/mL) compared to PH (39.2 ± 17.5 pg/mL) and LEX (1.8 ± 9.7 pg/mL). The back had a higher threshold than the forearm (*P* < 0.05) but it was similar to the chest (*P* > 0.05) (mean of all conditions; 0.64 ± 0.33, 0.42 ± 0.25, 0.54 ± 0.3 mg/cm^2^/min, respectively). Although the differences between conditions may be influenced by thermal or nonthermal mechanism, our results indicate a possibility that the sweat glands maximum ion reabsorption rates may be different between exercise and passive heating without mediating skin regional differences.

## Introduction

While sweating, humans excrete a number of different electrolytes in varying quantities across different regions on the body (Patterson et al. 2000). The quantity of these electrolytes, notably sodium chloride (NaCl), in sweat appears to be influenced by the sweat generation rate and the rate at which the ions are reabsorbed in the distal duct of the sweat glands (Shamsuddin et al. 2005b; Buono et al. 2007). Upon stimulation, the sweat glands produce an isosmotic precursor fluid in the proximal secretory coil (Shamsuddin et al. 2005b; Buono et al. 2007) that then travels toward the skin surface during which NaCl is reabsorbed in the distal duct, the result of which is a hypo‐osmotic fluid appearing on the skin surface (Allan and Wilson 1971; Sato 1977; Sato et al. 1989). The sweat rate (SR) at which sweat NaCl concentration increases rapidly is believed to reflect the point at which the maximum rate of ion reabsorption occurs (Shamsuddin et al. 2005a,b). Recent studies have identified the maximum rate of ion reabsorption in the context of skin temperature (Shamsuddin et al. 2005b), heat acclimation (Buono et al. 2007; Amano et al. 2016), long‐term exercise training status, sex and skin regions (Amano et al. 2017).

Previous studies have induced heat stress via either endogenous (exercise) or exogenous (passive heating) protocols, indicating maximum ion reabsorption rates, ranging between 0.19 and 0.83 mg/cm^2^/min (Shamsuddin et al. 2005a,b; Amano et al. 2016, 2017). The upper and lower ranges of these ion reabsorption rates have both been measured during exercise while passive heating protocols resulted in median ion reabsorption rates of approximately 0.3 mg/cm^2^/min. Such divergent results may be attributed to different experimental protocols employed such as ambient conditions, skin regions, and exercise protocols among previous studies. So it is unclear how the differences in exercise or passive heat stress would modify maximum rate of sweat ion reabsorption. Such data should help consolidate future experimental methodologies that may further attempt to understand the physiological mechanisms responsible for ion reabsorption rate of the sweat glands.

In comparison to a passive heating protocol, exercise induced thermal stress resulted in higher levels of water regulatory hormones (renin, aldosterone, and atrial natriuretic peptide) measured in blood plasma (Melin et al. 2001). The consequence of which was an enhanced renal tubular sodium reabsorption, which may have similar impacts on the ion regulatory mechanisms in the sweat glands (Melin et al. 2001; Hew‐Butler et al. 2010). In addition, it is also reported that the concentration of water regulating hormones are exercise intensity‐dependent (Freund et al. 1991; Yoshida et al. 2006) and Convertino et al. (1983) suggested that plasma renin activity and vasopressin increased above an approximate threshold of 50% *V*O_2max_. These increases may serve to enhance a greater water and NaCl retention and it is therefore thought that the ion reabsorption of the sweat glands during exercise and passive heating protocols could produce different responses, which would also be modulated by the exercise intensity.

Sweat rate and sweated ion concentrations are known to vary across the body and strong linear relationships are observed between the two (Patterson et al. 2000; Smith and Havenith 2012). However, the slopes of these relationships have been reported to differ among regions, which has been associated with the different reabsorption rates across sites (Inoue et al. 1998). The choice of locations for studying ion reabsorption rates seem to be at the discretion of the authors and have included the forearm (Amano et al. 2016), chest (Buono et al. 2007) and back (Shamsuddin et al. 2005a) with higher maximum ion reabsorptions rates reported at the torso in comparison to the extremities (Inoue et al. 1998; Amano et al. 2017). No data exist as to whether these regional difference vary with different heating protocols but it is unlikely given that NaCl concentration is correlated with local sweat gland output (Shamsuddin et al. 2005b; Buono et al. 2007) and previous studies suggest that regional differences in SR remain unchanged with increasing exercise intensity (Smith and Havenith 2011). In addition, regional differences in SR does not seem to be markedly different between exercise and passive heating (Taylor and Machado‐Moreira 2013).

The aim of this study was to compare maximum ion reabsorption rates of the sweat glands to passive heating, low intensity and moderate intensity exercise. Due to the differences in the fluid regulatory (renin and vasopressin) above and below 50% *V*O_2max_ reported previously (Convertino et al. 1983), it was hypothesized that that the maximum ion reabsorption rate of the eccrine sweat glands will be greater during moderate intensity exercise compare to both passive heating and low‐intensity exercise. In addition, it is hypothesized that the ion reabsorption rate will be similar between low‐intensity exercise and passive heating. To elucidate regional differences on the maximum rate of sweat ions reabsorption in the context of exercise and passive heating protocols, we evaluated the response across back, chest, and forearm. We further hypothesized that the ion reabsorption rates will be greater on the torso than the extremities in both conditions yet these regional differences will remain intact across the protocols.

## Methods

### Ethical approval

Participants were informed about the study purpose and procedures prior to providing verbal and written consent. The Human Subjects Committee of the Graduate School of Human Development and Environment at Kobe University (Japan) approved the study (report no. 259), which conforms to the standards set out by the Declaration of Helsinki (except for registration in a database).

### Participants

Males and females were recruited for this study, as our previous research indicated no sex‐related differences in ion reabsorption rates (Amano et al. 2017). Twelve un‐acclimated participants (five females and seven males) were recruited for the study. Mean (± SD) age, body mass, height, and *V*O_2max_ were 21.7 ± 3.0 years, 59.4 ± 9.8 kg, 166.9 ± 10.4 cm, and 47.1 ± 7.5 mL/kg/min. Participants were asked to refrain from consuming high sodium foods, caffeine or alcohol and to avoid any strenuous exercise 24 h preceding the trials. For the experimental trials they were instructed to record their food and beverage intake during the preceding 24 h and asked to replicate this for the remaining trials. To promote euhydration, participants were instructed to consume at least 500 mL of water 1–2 h prior to experiment. All participants were nonsmokers and were not taking any medications. Menstrual cycle phase was not controlled for in the female participants.

### Study overview

Prior to the main experimental trials participants completed a maximal oxygen uptake test on a cycle ergometer. Participants were then required to visit the laboratory a further 3 times for the main experimental trials that were separated by at least 48 h. The experimental trials consisted of two separate endogenous (cycling exercise) trials and one exogenous heating trial (passive heating), in a balanced order. All experimental trials were conducted in a climatic chamber (SR‐3000; Nagano Science, Osaka, Japan) controlled at 27°C, 50% relative humidity, with minimal air movement. All tests were completed at the same time of day (± 1.5 h), at least 2 h after their last meal. During all experimental trials, participants wore standardized shorts and females also wore a sports bra underneath a water‐perfused suit (Allen‐Vanguard, Ottawa, Canada) to help elevate the thermal load and in an attempt to maintain a similar skin temperature across the body.

### Graded exercise test

Utilizing a ramp protocol, participants completed a 2‐min warm up at 20W, 60 rpm, followed by a continual increase in 20W every minute until exhaustion on a semirecumbent cycle ergometer (Lode Angio, Groningen, The Netherlands). Respiratory gases were continuously monitored throughout using an online gas analysis system (AE3000S; Minato Medical Science, Osaka, Japan) with 10‐sec averages being recorded. Heart rate was continuously monitored by telemetry using a HR monitor (Polar RS400; Polar Electro Oy, Kempele, Finland) and 10‐sec averages were recorded. The *V*O_2max_ was calculated as the average oxygen consumption over the final 30 sec. Testing took place in a controlled environment of 25°C, 50% RH and minimal air movement.

### Experimental trials

Upon entering the chamber participants donned a water‐perfused suit that covered the entire body except face, hands, and lower legs (from above the knee). Participants then rested in a semi supine position for approximately 60 min while the measuring instruments were attached (detailed below). During the instrumentation preparation phase, water was passed through the suit at 34°C to maintain a stable resting skin temperature. Following instrumentations, baseline data were recorded for 5 min. Participants were then heated either endogenously or exogenously. Exogenous heating involved immersing the lower legs (up to the knee) into a water bath set at 43°C for 30 min (passive heating, hereafter abbreviated to PH). Endogenous heating trials were performance at either 30% (low intensity exercise, hereafter abbreviated to LEX) or 60% (moderate intensity exercise, hereafter abbreviated to MEX) of *V*O_2max_ for 30 min on the same semi recumbent cycle ergometer used for the graded exercise test. The same chair was used for PH, LEX and MEX and therefore the same posture was maintained for all conditions. During the heating phase in all experimental trials, the water temperature inside the suit was increased to 36°C to maintain stable yet elevated skin temperature.

### Measurements

Participants self inserted the esophageal thermometer, as an index of core temperature (*T*_es_) to the distance of one‐fourth of standing height from the external nares. Skin temperature (*T*_sk_) was measured at 9 sites (forehead, chest, scapula, lumbar, upper posterior arm, posterior forearm, dorsal hand, anterior thigh, and posterior calf). Both the oesophageal and skin temperatures were measured using copper‐constantan thermocouples (Inui Engineering, Higashi Osaka, Japan). The tip of the esophageal thermometer was covered with silicon and the skin thermocouples were uncovered and attached to the skin using Medipore tape. Mean skin temperature (from 8 sites) and mean body temperature (*T*_b_) was calculated using the following respective formula (Stolwijk and Hardy 1966; Gagge and Nishi 2011);

$$\begin{array}{cc} \text{Mean}\;T_{\mathrm{sk}}= & \left(\text{forehead}\times 0.07)+(\text{chest}\times 0.175\right)\\ & +\left(\text{scapula}\times 0.175\right)+\left(\text{upper posterior arm}\right.\\ & \times 0.07\left.\right)+\left(\text{posterior forearm}\times 0.07\right)+\left(\text{hand}\right.\\ & \times 0.05\left.\right)+\left(\text{anterior thigh}\times 0.19\right)\\ & +(\text{posterior calf}\times 0.2) \end{array}$$

$$T_{b}=\left(0.8\times T_{\mathrm{es}}\right)+\left(0.2\times \text{mean}\;T_{\mathrm{sk}}\right)$$

SR was measured using the ventilated capsule method at the mid‐chest, lower back to the right side of the lumbar spine and mid‐ventral forearm, all on the right side of the body. Dry nitrogen gas was flushed (500 mL/min) through the apparatus approximately 1 h prior to each experiment to ensure stable readings. Each capsule (3.14 cm^2^) was affixed to the skin using Collodion glue at least 30 min prior to data collection. The temperature and humidity of the air flowing out of the capsule was measured using a capacitance hygrometer (HMP50; Vaisala, Helsinki, Finland). Two Ag/AgCl electrodes (Vitrode J, Nihon Kohden, Tokyo, Japan) for measuring GSC were attached either side of the sweat capsules, approximately 3 cm apart (MP100 and GSC100C; Biopac, Goleta CA). Both the SR capsule and the Ag/AgCl electrodes were placed beneath the suit. *T*_es_, local *T*_sk_, SR and GSC were recorded every second by a data logger (MX100; Yokogawa, Tokyo, Japan). Heart rate and arterial blood pressure were continuously measured on the left middle finger using a Finometer (Finometer; Finapres Medical Systems, Amsterdam, The Netherlands); mean arterial pressure (MAP) was subsequently calculated.

### Measuring the sweat glands maximum ion reabsorption rate

**Figure 1:**
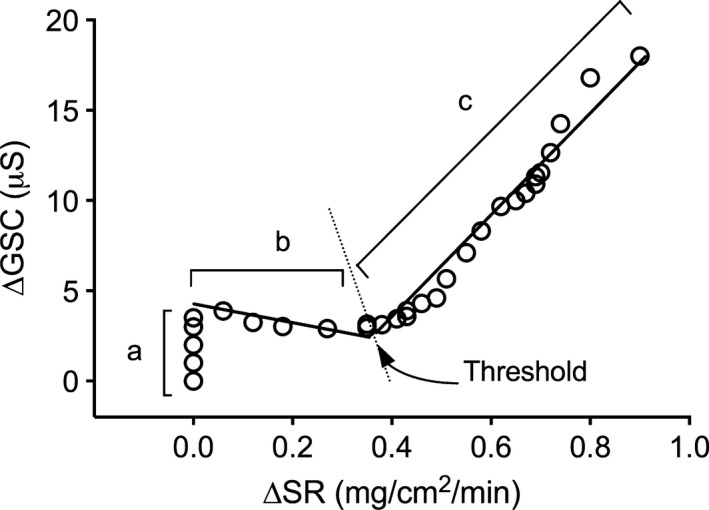
Example of the relationship between changes in galvanic skin conductance (∆GSC) and sweat rate (∆SR) in one participant.

As described in our previous experiment (Amano et al. 2016), the maximum reabsorption rate of the sweat glands can be obtained by plotting ΔGSC against ∆SR as shown in Figure 1. By plotting this relationship, it is possible to identify three distinct phases; representing different stages of sweat production. In the first phase, labeled “a” there is an increase in ∆GSC but no change in ∆SR, which represents the isosmotic precursor sweat production in the proximal secretory coil. Such changes in ∆GSC and no changes in ∆SR are frequently utilized to identify pre‐secretory sweat gland activity (Thomas and Korr 1957; Darrow 1964; Machado‐Moreira et al. 2009). In the second phase, labeled “b” an increased ∆SR without an increase in ΔGSC can be observed. As ∆GSC is influenced by both the amount of sweat produced as well as the electrolyte concentration the fact that ∆SR increases but there is no change in ∆GSC represents reabsorption of sweated ions in the sweat duct. Once the rate of sweat ion secretion exceeds its reabsorption limit in the duct then a proportional increase in ΔGSC with increasing ∆SR can be observed, labeled “c”. The point at which “b” and “c” intersect is used to identify the maximum rate of sweat glands ion reabsorption. In this study, the threshold between portion “b” and “c” were determined using segmented regression analysis on GraphPad Prism (version 6) software.

### Follow up study –salivary aldosterone

In a small follow‐up study, three participants repeated the three conditions while salivary aldosterone samples were collected at rest and at the end of each condition (LEX, MEX, and PH). Previous research has reported strong correlations between saliva, plasma, and urine aldosterone concentrations (Robert Mcvie and Levine 1979; Few et al. 1986; Manolopoulou et al. 2009). As per the main trial, participants were asked to refrain from consuming high‐sodium foods, caffeine or alcohol and to avoid any strenuous exercise 24 h preceding the trials and a minimum of 48 h separated trials. They were also requested to consume 500 mL of water 1–2 h before each trial. Participants rested in the environmental chamber (27°C, 50%RH) for 30 min prior to baseline sampling. During the final minute of each condition a salivary sample was taken. Salivary aldosterone was collected using Salivettes™ (Sarstedt, Newton, NC) whereby a plain cotton swab was inserted into the mouth and chewed for 60 sec. The cotton swab was then returned into the Salivette™ tube and spun at 4000RPM for 10 min. Samples were then frozen at −30°C until analysis. After thawing, salivary aldosterone (pg/mL) levels were quantified by competitive ELISA (LDN, GmbH & Co.KG, Germany). The sensitivity of the assay for aldosterone was 14 pg/mL and the inter‐ and intra‐assay coefficient of variation were between 3.9% and 7.5% and 9.4–9.7%, respectively.

### Data analysis

To investigate the effect of exogenous (passive) and endogenous (exercise) heating on physiological parameters (local maximum ion reabsorption) a two‐way repeated measure ANOVA with condition (PH, LEX and MEX) and location (chest, forearm and back) as repeated measures was carried out. As time was not a factor in our hypothesis, the area under the time response curve as a measure of the cumulative (or equivalent, mean) response was calculated for local *T*_sk_, ∆SR and ∆GSC at the three measurement sites and subsequently used in a two‐way ANOVA to analyse the effect of condition (PH, LEX and MEX) and location (forearm, chest and back), otherwise known as *within subject modeling* (Hopkins et al. 2009). Two‐way repeated measures ANOVA with time (7 levels: BL, 5, 10, 15, 20, 25, 30 min) and condition (3 levels: PH, LEX and MEX) was used to assess the remaining thermophysiological (*T*_es_, *T*_b_ and mean *T*_sk_) and cardiovascular responses (HR and MAP). When any significant effects were observed, post hoc comparison using the Bonferroni test were carried out. All data were checked for homogeneity of variance and normality (Shapiro‐Wilk test). Both HR and MAP violated these assumptions, so a Friedman's test was performed (comparing LEX, MEX, and PH data). All data are expressed as means and standard deviations and statistical significance was set at *P* < 0.05. All data were analyzed using GraphPad Prism (version 6).

## Results

### Thermo‐physiological measurements during the heating protocols

The influence of each heating protocol on thermoregulatory responses; *T*_es_, *T*_b_, and mean *T*_sk_ are displayed in Table 1. For *T*_es_, statistical analysis revealed a main effect of condition and post hoc analysis revealed that *T*_es_ was higher during MEX than PH and LEX (*P* < 0.05) but there were no differences between PH and LEX (*P* > 0.05). A main effect of time was observed with all data from 10 to 30 min being higher than baseline. An interaction effect was also observed and post hoc comparisons are highlighted in Table 1. A main effect of condition was also observed for mean T_sk_ and post hoc analysis revealed that mean *T*_sk_ was lower during LEX compared to PH and MEX (*P* < 0.05), and PH was higher than MEX (*P* < 0.05). A main effect of time was also observed with all data points from 10 to 30 min being higher than baseline (*P* < 0.05). An interaction effect was also observed and post hoc comparisons are highlighted in Table 1. A main effect of condition was observed for *T*_b_ and post hoc analysis revealed that *T*_b_ was lower during LEX compared to PH and MEX (*P* < 0.05) but there were no differences between PH and MEX (*P* > 0.05). A main effect of time was also observed with all data from 10 to 30 min being higher than baseline (*P* < 0.05). An interaction effect was also observed and post hoc comparisons are highlighted in Table 1.

**Table 1:** Thermophysiological and cardiovascular parameters during low‐intensity exercise (LEX), moderate intensity exercise (MEX) and passive heating (PH) at baseline (BL) and at 5‐min stages throughout each protocol

		BL	5 min	10 min	15 min	20 min	25 min	30 min	Sign
T_es_ (˚C)	LEX	36.9 ± 0.2	36.8 ± 0.2#	37.0 ± 0.2*, #	37.1 ± 0.2*, $, #	37.1 ± 0.2*, $, #	37.2 ± 0.2*, $, #	37.2 ± 0.2*, $, #	# ϕ
	MEX	36.9 ± 0.3	37.0 ± 0.3#	37.5 ± 0.4*, #, ϕ	37.7 ± 0.4*, #, ϕ	37.9 ± 0.5*, #, ϕ	38.1 ± 0.6*, #, ϕ	38.3 ± 0.7*, #, ϕ	
	PH	36.9 ± 0.4	36.9 ± 0.4	37.1 ± 0.4*, ϕ	37.5 ± 0.4*, $, ϕ	37.4 ± 0.4*, $, ϕ	37.5 ± 0.4*, $, ϕ	37.6 ± 0.4*, $, ϕ	
T_b_ (˚C)	LEX	36.3 ± 0.2	36.2 ± 0.3$	36.3 ± 0.5*, $, #	36.4 ± 0.4*, $, #	36.5 ± 0.4*, $, #	36.5 ± 0.4*, $, #	36.6 ± 0.4*, $, #	$ #
	MEX	36.4 ± 0.4	36.3 ± 0.4ϕ	36.7 ± 0.7*, #	37.0 ± 0.7*, #	37.2 ± 0.8*, #	37.4 ± 0.8*, #	37.5 ± 0.9*, #	
	PH	36.4 ± 0.4	36.7 ± 0.3ϕ	37.0 ± 0.4*, $	37.2 ± 0.4*, $	37.4 ± 0.4*, $	37.5 ± 0.3*, $	37.5 ± 0.3*, $	
Mean T_sk_ (˚C)	LEX	34.2 ± 0.5$	34.1 ± 0.5$	34.3 ± 0.6*, $	34.3 ± 0.6*, $	34.4 ± 0.6*, $	34.4 ± 0.6*, $	34.5 ± 0.5*, $	$ # ϕ
	MEX	34.2 ± 0.5	34.2 ± 0.4ϕ	34.6 ± 0.6*, #, ϕ	35.1 ± 0.6*, #, ϕ	35.3 ± 0.5*, #, ϕ	35.5 ± 0.5*, #, ϕ	35.5 ± 0.5*, #, ϕ	
	PH	34.4 ± 0.5$	35.9 ± 0.5$, ϕ	36.5 ± 0.6*, $, ϕ	36.8 ± 0.7*, $, ϕ	37.0 ± 0.6*, $, ϕ	37.1 ± 0.4*, $, ϕ	37.2 ± 0.4*, $, ϕ	
HR (bpm)	LEX	63.3 ± 10.7	91.5 ± 10.4*, $, #	94.3 ± 11.5*, #	96.8 ± 10.7*, #	98.8 ± 11.3*, #	98.6 ± 11.5*, #	99.4 ± 11.8*, #	$ # ϕ
	MEX	64.5 ± 9.6	116.1 ± 13.8*, ϕ	133.7 ± 10.3*, ϕ	144.8 ± 12.1*, ϕ	148.2 ± 12.3*, ϕ	150.4 ± 12.2*, ϕ	151.9 ± 11.2*, ϕ	
	PH	64.8 ± 10.8	73.6 ± 16.4$, ϕ	80.5 ± 22.1ϕ	85.4 ± 23.3*, ϕ	88.7 ± 22.3*, ϕ	91.1 ± 20.9*, ϕ	93.4 ± 20.3*, ϕ	
MAP (mmHg)	LEX	92.3 ± 9.5	98.3 ± 7.8#	100.2 ± 8.6#	99.5 ± 9.3#	99.3 ± 9.0#	99.5 ± 8.0#	99.2 ± 8.5#	$ # ϕ
	MEX	93.2 ± 8.0	120.5 ± 14.7*, #, ϕ	116.8 ± 11.7*, #, ϕ	111.8 ± 10*, #, ϕ	111.7 ± 9.5*, #, ϕ	112.0 ± 10.9*, #, ϕ	112.3 ± 10.3*, #, ϕ	
	PH	93.8 ± 8.8	97.7 ± 11.9ϕ	98.6 ± 13.1ϕ	96.2 ± 12.1ϕ	94.2 ± 11ϕ	94.1 ± 10ϕ	95.1 ± 9.9ϕ	

Values are means ± SD for 12 participants measured at baseline (BL) and over 5 minute periods during three different conditions; LEX, low‐intensity exercise, MEX, moderate intensity exercise; PH, passive heating. HR, heart rate; MAP, mean arterial pressure. Two‐way ANOVA revealed a main effect of condition and time for all variables presented in this table. Post hoc comparison for condition is indicated by the following: ^$^for LEX vs. PH, ^#^ for LEX vs. MEX, and ^*ϕ*^ for MEX vs. PH (all *P* < 0.05). Post hoc comparisons of time are indicated by *(*P* < 0.05), which shows where the time point was higher than baseline (BL). An interaction effect was observed between time and condition for all variables as indicated by ^$^ for LEX vs. PH, ^#^ for LEX vs. MEX, and ^*ϕ*^ for MEX vs. PH (all *P* < 0.05).

**Figure 2:**
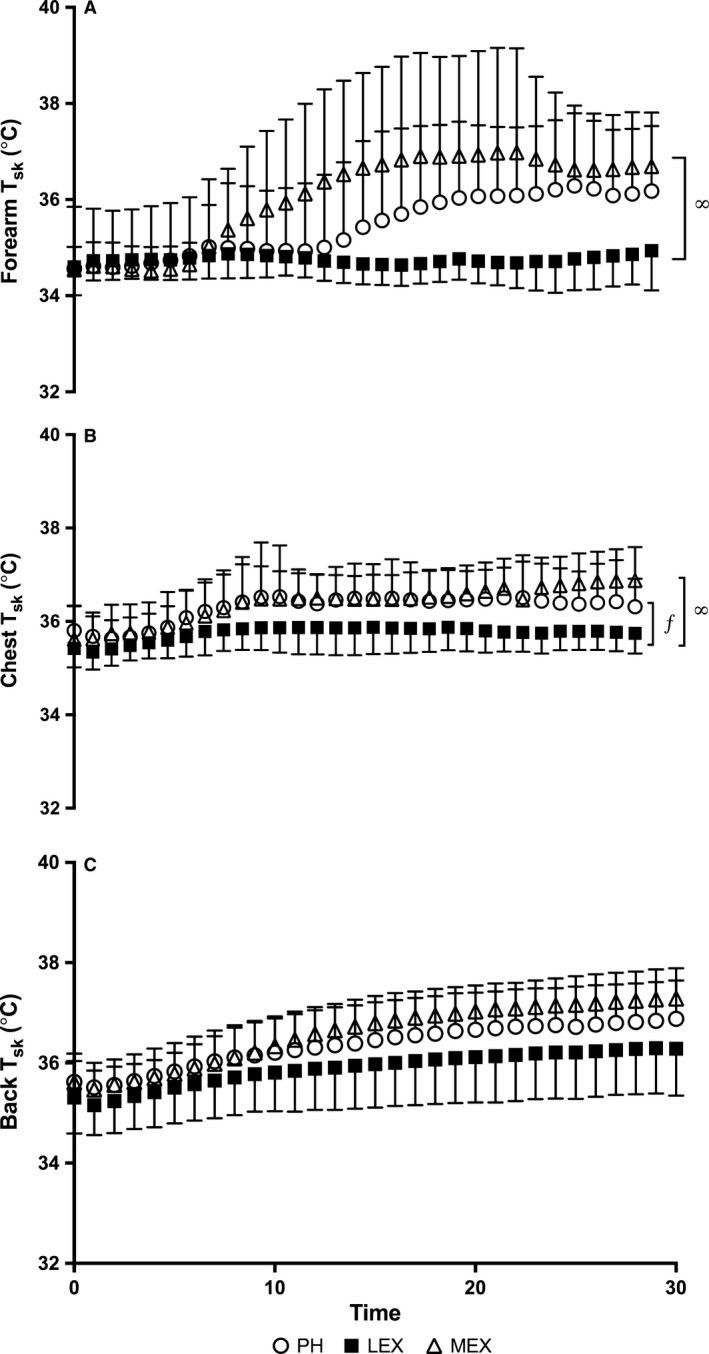
Local skin temperature (*T*_sk_) at the forearm (*A*), chest (*B*) and back (*C*) measured over time during passive heating (PH), low‐intensity exercise (LEX) and moderate intensity exercise (MEX). Symbols *, ∞ and ƒ indicates significant differences between PH and MEX, LEX and MEX and LEX and PH, respectively (*P* < 0.05). Values are expressed as mean ± SD for 12 participants.

Skin temperature measured at the three locations under investigation for ion reabsorption rates (forearm, chest, and back) are illustrated in Figure 2. Using the area under the curve, a main effect of condition and location was observed (*P* < 0.05). Post hoc analysis revealed that cumulative effect (i.e., the AUC) for the forearm *T*_sk_ was smaller during LEX compared to MEX and PH (4.5, 45.5, and 26.3°C, respectively *P* < 0.05) but no differences were observed between PH and MEX (*P* > 0.05). A smaller cumulative effect for the chest *T*_sk_ was observed during LEX compared to MEX (10.2 and 23.1°C, respectively, *P* < 0.05) but it was not different between LEX and PH (15.6°C) or between PH and MEX (*P* > 0.05). Back *T*_sk_ was similar between conditions.

**Figure 3:**
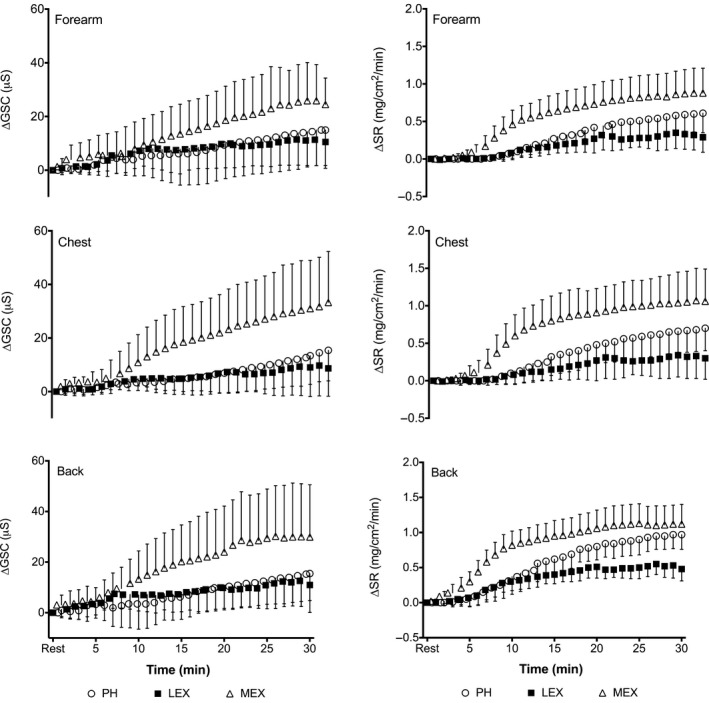
Left column shows local ∆GSC and right column shows ∆SR during passive heating (PH), low‐intensity exercise (LEX) and moderate intensity exercise (MEX) measured at the forearm, chest and back. Values are expressed as mean ± SD for 12 participants.

Local ∆GSC and ∆SR over time in each of the three conditions are presented in Figure 3. A main effect of condition and location were observed for the AUC of the ∆SR data. ∆SR was greater during MEX compared to PH and LEX (20.6, 11.9, and 6.9 mg/cm^2^, respectively, *P* < 0.05). All locations were significantly different to one another, with the largest AUC measured at the back, chest and forearm (16.9, 12.1, and 10.3 mg/cm^2^, respectively, *P* < 0.05). For ∆GSC a main affect of condition was found with a larger AUC reported for MEX (501.4 *μ*S) compared to both PH (210.9 *μ*S) and LEX (204.9 *μ*S) (*P* < 0.05). No effect of location was detected (*P* > 0.05).

### ∆Sweat rate threshold for an increasing ∆GSC

**Figure 4:**
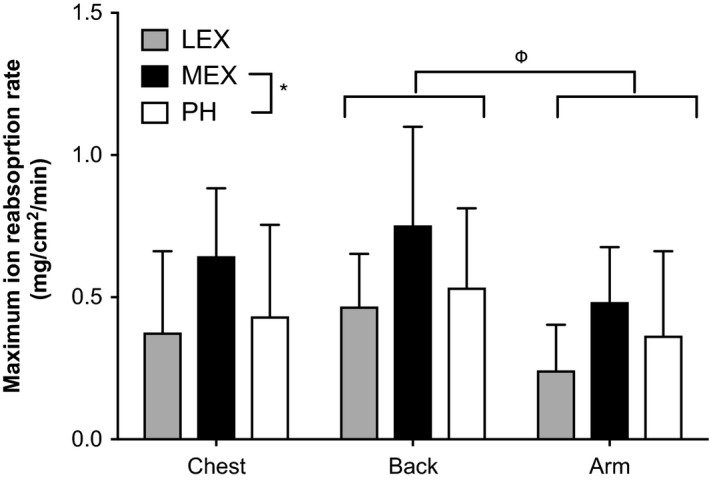
The **∆** SR threshold for an increasing ∆GSC during low‐intensity exercise (LEX) moderate intensity exercise (MEX) and passive heating (PH) at the forearm, chest and back. Values are expressed as mean ± SD for 12 participants for all MEX and PH data, however, LEX at the chest and forearm are the mean ± SD for 8 participants and the back is the mean ± SD for 7 participants**.** Therefore, LEX data were excluded from statistical analysis. MEX was higher than PH (*^,^ *P* < 0.05) and the back had a higher reabsorption rate than the forearm (indicated by ^Φ,^ *P* < 0.05) but there was no interaction effect. LEX, low‐intensity exercise; MEX, moderate intensity exercise; PH, passive heating**.**

The mean (±SD) values for the sweat rate threshold for an increasing ∆GSC are presented in Figure 4.

During LEX, it was not always possible to identify the ∆SR threshold for an increasing ∆GSC suggesting that the maximum reabsorption rate had not been reached. This occurred for three participants at all three locations, while in other participants it occurred at only one or two locations. This resulted in collected data at the forearm, chest, and back from 8, 8, and 7 participants (respectively). As these were not necessarily the same participants at each location, it resulted in only five full data sets (all three conditions and all three locations). This implicated the data analysis and it was therefore deemed appropriate to only statistically compare PH and MEX ion reabsorption data. Analysis revealed an effect of condition and location (*P* < 0.05) but no interaction (condition x location) (*P* > 0.05). The maximum ion reabsorption rate occurred at a higher ∆SR during MEX compared to PH (mean of all data; 0.63 ± 0.28 and 0.54 ± 0.30 mg/cm^2^/min, respectively, *P* < 0.05). An effect of location was also observed with the back having a higher maximum reabsorption rate than the forearm (mean of all data: 0.64 ± 0.33 and 0.42 ± 0.25 mg/cm^2^/min, respectively, *P* < 0.05) but the chest (0.54 ± 0.30 mg/cm^2^/min) was not different to either the back or forearm (*P* > 0.05).

Although LEX was excluded from the statistical analysis the remaining data can be used in an exploratory manner to observe any trends. From Figure 4 it is evident that the sweat rate threshold for an increasing ∆GSC is considerably lower during LEX compared to MEX (mean difference −0.36 mg/cm^2^/min) and slightly lower than PH (mean difference −0.18 mg/cm^2^/min). In addition, the back had a higher maximum ion reabsorption rate than both the chest and the forearm, while the forearm and chest were very similar.

**Figure 5:**
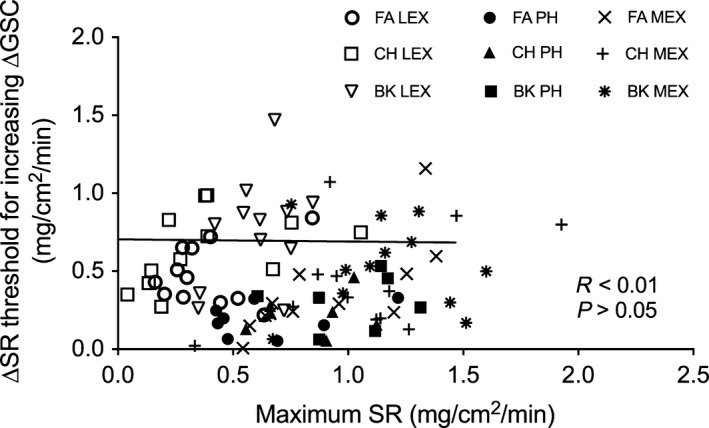
Sweat rate (SR) thresholds for an increasing ∆GSC on the forearm (FA), chest (CH) and back (BK) plotted against the maximum SR achieved during low‐intensity exercise (LEX), moderate intensity exercise (MEX) and passive heating (PH).

As per our previous study, we plotted the relation between ∆SR thresholds for an increasing ∆GSC against the maximum SR (see Fig. 5) at the end of each protocol (inclusive of all three locations and all three conditions) and found no relation (*r* ^2^<0.001, *P* > 0.05). This was still the case when we separated the data for each condition (combining the three locations) and when we grouped the data according to each location (combining the three conditions). We found a strong significant relation at the forearm only (*r* ^2^= 0.77, *P* < 0.05).

### Cardiovascular measurements during the heating protocols

Cardiovascular responses to the three different protocols are displayed in Table 1. A significant effect of condition were present for HR and MAP (*P* < 0.001). HR and MAP were lower during PH and LEX compared to MEX (*P* < 0.001) and LEX was lower than PH (*P* > 0.001).

### Salivary aldosterone

Salivary aldosterone levels showed the greatest increase from baseline to the end of MEX (∆72.8 ± 35.6 pg/mL, range: 31.7–94.6 pg/mL). An increase was also observed during PH (∆39.2 ± 17.5 pg/mL, range 22.1–57.0 pg/mL) and a very small change observed during LEX (∆1.8 ± 9.7 pg/mL, range −5 to 12.85 pg/mL).

## Discussion

### Influence of endogenous and exogenous heating

The ∆SR threshold for an increasing ∆GSC has recently been proposed as a method of assessing the sweat glands maximum ion reabsorption rate (Amano et al. 2016). The main finding from this study was that endogenous and exogenous heating may have different effects on the sweat gland(s) ion reabsorption rates. It has been suggested that Na^+^ secretion increases proportionally faster than the reabsorption rate with an increasing sweat rate, hence why a linear relationship between SR and sweated ion concentration can be observed (Buono et al. 2008). While this may be the case, our data suggests that the ion reabsorption rate can be altered. Endogenous heating resulted in a higher ∆SR threshold for an increasing ∆GSC, indicating a higher maximum reabsorption rate than exogenous heating at all three locations. The mechanism by which this is achieved may be associated with thermal or nonthermal mechanisms associated with the different protocols. Importance of thermal mechanism such as *T*_es_ and *T*_sk_ has previously been suggested (Shamsuddin et al. 2005a). During PH, *T*_sk_ of the lower leg mirrored water temperature of the immersion bath (43°C), resulting in a higher mean *T*_sk_ compared to MEX, whereas a greater rise in *T*_es_ was observed during MEX. As a result of the different protocols used, the differences in skin and core temperature were inevitable but we were able to match *T*_b_ and local *T*_sk_ at the 3 measured locations (forearm, chest, and back). Given that *T*_c_ has a greater influence on sweat rate compared to *T*_c_ (Nadel et al. 1971) it is possible that the differences in the maximum ion reabsorption rate observed between the two conditions may result from a higher *T*_c_ during MEX compared to PH. By plotting the maximum SR against the maximum ion reabsorption rate in our previous experiment (Amano et al. 2017) we showed that SR may explain 75% of the variance in ion reabsorption rate during a progressively increasing exercise intensity protocol. We replicated this analysis in this study and we found no relation (see Fig. 5). The aim of this study was to investigate if different heating protocols affected the maximum ion reabsorption rate. There would be no clear relationship if differences occurred, as we hypothesized. This may provide additional evidence for the influence of heating protocols on ion reabsorption rates of the sweat glands but further research is required to confirm this.

The influence of *T*_sk_ on the ion reabsorption rate of the sweat glands was previously investigated by Shamsuddin et al. (2005a), whereby lowering mean *T*_sk_ (∆mean*T*_sk_ ~3.0°C) via changing ambient conditions (19°C vs. 25°C) during exercise inhibited the ion reabsorption capacity of the sweat gland. Their results conflict with this study whereby Shamsuddin et al. (2005a) reported a lower ion reabsorption rate for a lower mean *T*_sk_. However, in this study, mean *T*_sk_ during MEX was lower than PH yet the ion reabsorption rate was higher. This contrasting finding may lean more toward nonthermal mechanisms (see following discussion) for the differences observed between endogenous and exogenous heating protocols in our study. In addition, Shamsuddin et al. (2005a) reported the differences in mean *T*_sk_ and not the local *T*_sk_ at the measured location. Our data showed differences in mean *T*_sk_ which was primarily due to differences in lower leg *T*_sk_ mirroring water bath temperature (43°C), while local T_sk_ at the chest, back, and forearm (and at other locations) were not significantly different between the two conditions.

If our thermophysiological responses had been matched between the two conditions, we hypothesize that the differences observed between the maximum ion reabsorption rate would still be evident, primarily due to the sympathetic differences between such protocols. Studies aimed to separate the thermal and exercise effects on various physiological responses have documented differences in sympathetic nervous system and hypothalamic‐pituitary‐adrenal‐axis responses (Collins and Weiner 1968; Francesconi 1988; Brenner et al. 1998). Fluid regulatory hormones have too been shown to differ between endogenous and exogenous heating protocols with elevated levels of plasma renin and aldosterone and also plasma catecholamines during exercise compared to passive heating (Melin et al. 2001). In a small follow up study we measured pre‐ and postsalivary aldosterone concentrations during each protocol (*n* = 3). Larger increases in salivary aldosterone were observed during MEX (∆72.8 ± 36.6 pg/mL) compared to both PH (∆39.1 ± 17.5 pg/mL) and LEX (∆1.8 ± 9.7 pg/mL). Aldosterone is known for its role in regulating sodium reabsorption in the distal segment of the nephron in the kidneys, which has been likened to the reabsorptive distal duct of the sweat gland that also contains epithelial sodium channel (ENaC) and cystic fibrosis transmembrane regulator (CFTR) that are regulated by the renin‐angiotensin‐aldosterone axis, anti‐diuretic hormones and catecholamines (Alvarez De La Rosa et al. 2000; Reddy and Stutts 2013). The rapid nongenomic action of aldosterone selectively targets basolateral K^+^ pump and Na^+^/H^+^ exchanger in distal human sweat glands via the activation of protein kinase C (PKC) and calcium signaling (Hegarty and Harvey 1999; Harvey and Higgins 2000). The result of which is the promotion of sodium transport from apical to basolateral spaces (i.e., sodium reabsorption). Harvey and Higgins (2000) reported changes in aldosterone as small as 0.1 nmol/L (or 36 pg/mL) significantly increased intracellular Ca^2+^ transporters in mouse cortical collecting duct, which subsequently activated Na^+^/H^+^ exchanger. Thus, our aldosterone data might suggest a greater humoral regulation of the sweat ions reabsorption rate during MEX compared to PH although this needs to be confirmed in matched thermal conditions. Alternatively, it is also reported that other non‐thermal mechanisms such as the activity of the fluid and sodium regulatory channels lining the sweat glands (e.g., aquaporin‐5 water channel and co‐activated CFTR and the ENaC may play a role for regulating sweat sodium reabsorption; Reddy and Quinton 1994, 2003; Brown et al. 2011; Bovell 2015). It is however, unknown whether and how they affected the differences in the maximum rate of sweat reabsorption between PH and MEX in this study.

LEX data were excluded from the statistical analysis, as it was not possible to identify the maximum ion reabsorption at some location for some participants. For these participants, it is simply assumed that the level of sweating during LEX was too low to identify the threshold and in such cases, when sweat rate is low, the ion reabsorption rate can match the ion secretion rate. The remaining data indicated that there was a trend for a lower threshold at all locations during LEX compared to PH and MEX. Plasma renin activity and vasopressin have been shown to increase above an approximate threshold of 50% *V*O_2max_ which enhances water and NaCl retention during exercise (Convertino et al. 1983). The exercise intensity during LEX corresponded to 30% *V*O_2max_ while MEX corresponded to 60% *V*O_2max_. While speculative, it is therefore assumed that potential hormone secretion during exercise at 30% *V*O_2max_ might be varied among individuals which might make it difficult to identify the threshold for some of our participants in this study. In support of this the aldosterone levels remained unchanged during LEX (∆1.8 ± 9.7 pg/mL). Furthermore, for three participants it was not possible to identify the threshold at any of the three locations during LEX but in some participants it was possible to identify the threshold at one or two locations, which supports the hypothesis of regional differences in the ion reabsorption rates.

### Regional differences

Patterson et al. (2000) reported regional differences (10 sites) in sweat rate and sweat composition and found strong relationships between Na^+^ and Cl^−^ with sweat rate for individual participants and at individual locations but when the data were pooled for all participants and all locations poor correlation coefficients were observed. They suggested that this could be attributed to individual and regional differences in ion reabsorptive capacity of the sweat gland(s). In this study, regional differences in the maximum ion reabsorption rates were observed (high to low) at the back, chest and the forearm. Data analysis revealed that the back was significantly higher than the forearm. This data supports our previous research whereby the back has a higher ion reabsorption rate than the forearm (Amano et al. 2017) and the torso ion reabsorption rate being greater than the extremities (Inoue et al. 1998). We can now confirm that these regional differences are still apparent during both endo‐ and exogenous heating protocols. In addition, while speculative, this study also implies that potential nonthermal influences (e.g., hormones) would equally affect the maximum rate of sweat reabsorption across the body regions.

Amano et al. (2017) reported similar regional differences in ion reabsorption rates but these could not attributed to any potential mechanistic differences, as local *T*_sk_ was reportedly different between sites. In this study regional differences in *T*_sk_ were not apparent between the forearm, chest and back during MEX but the forearm *T*_sk_ was lower than the back during PH. During MEX the ion reabsorption rate of the forearm was significantly lower (0.57 ± 0.28 mg/cm^2^/min) than the back (0.99 ± 0.53 mg/cm^2^/min), which in this data cannot be explained by differences in regional *T*_sk_. The torso, especially posterior torso has consistently been reported as having greater sweat rate in comparison to the anterior torso and the extremities (Cotter et al. 1995; Havenith et al. 2008; Smith and Havenith 2011, 2012) and thus a potentially larger sweated ion loss. In order to prevent excessive ion loss from the torso region, having a higher reabsorption rate could be a teleological conception that would be advantageous for maintaining fluid and electrolyte balance. We previously proposed that irrespective of regional differences, sex and exercise training, sweat gland(s) that can produce larger volumes of sweat have a greater capacity of sweat ion reabsorption as indicated by the strong significant relationships observed between the ∆SR threshold for an increasing ∆GSC with measured SR at the end of exercise (Amano et al. 2017). However, in this study this relationship was not observed which might be due to the differing protocols of the two studies. The regional differences observed may be influenced by structural differences in the sweat glands, such as a longer reabsorptive duct, greater capillarization around the sweat gland for the delivery of fluid regulatory hormones, or sudomotor sensitivity might account for the differences in ion reabsorptive and sweated ion concentrations reported here and elsewhere.

### Perspectives and significance

The physiological benefit of having a higher ion reabsorption rate is the release of more dilute sweat onto the skin surface via the attenuation of ion loss (namely NaCl). As such, in the event of prolonged sweat production, decreases in plasma volume and increases in plasma osmotic pressure will both be attenuated (Senay 1968). Given the linear relationship observed between sweat rate and sweated Na^+^ concentration (Buono et al. 2008), if sweat rate is high then so too will be the secreted ions. In this study, sweat rate at all three locations was greater during MEX in comparison to PH and LEX. Therefore, we speculate that in order to prevent excessive sweated ion loss that can lead to reductions in plasma volume and heightened cardiovascular strain, the maximum ion reabsorption rate of the sweat glands can be regulated and this may be mediated by thermal and nonthermal modulators such as aldosterone. Future studies are required to investigate conditions where *T*_c_ is matched between passive and active heating protocols.

## Conclusion

Endogenous heating resulted in a higher ∆SR threshold for an increasing ∆GSC, indicating a higher maximum reabsorption rate than exogenous heating at the chest, back and forearm. As this difference may be influenced by either thermal or nonthermal mechanisms future research is required to address these points independently. Furthermore, independent of thermal influences, typical regional differences remained intact despite the differences in heating protocols. We provide important insights for future research studies and alluding to potential controlling mechanisms for understanding the sweat ion regulation at the level of the sweat gland.

## Conflict of Interest

None declared.
